# Indigenous sex-selective salmon harvesting demonstrates pre-contact marine resource management in Burrard Inlet, British Columbia, Canada

**DOI:** 10.1038/s41598-021-00154-4

**Published:** 2021-11-10

**Authors:** Jesse Morin, Thomas C. A. Royle, Hua Zhang, Camilla Speller, Miguel Alcaide, Ryan Morin, Morgan Ritchie, Aubrey Cannon, Michael George, Michelle George, Dongya Yang

**Affiliations:** 1 Institute for the Oceans and Fisheries, University of British Columbia, Vancouver, BC Canada; 2 Ancient DNA Laboratory, Department of Archaeology, Simon Fraser University, Burnaby, BC Canada; 3 Department of Anthropology, University of British Columbia, Vancouver, BC Canada; 4 Department of Molecular Biology and Biochemistry, Simon Fraser University, Burnaby, BC Canada; 5 Department of Anthropology, McMaster University, Hamilton, ON Canada; 6 Tsleil-Waututh Nation, North Vancouver, BC Canada

**Keywords:** Sustainability, Conservation biology, Ichthyology

## Abstract

To gain insight into pre-contact Coast Salish fishing practices, we used new palaeogenetic analytical techniques to assign sex identifications to salmonid bones from four archaeological sites in Burrard Inlet (*Tsleil-Waut*), British Columbia, Canada, dating between about 2300–1000 BP (ca. 400 BCE–CE 1200). Our results indicate that male chum salmon (*Oncorhynchus keta*) were preferentially targeted at two of the four sampled archaeological sites. Because a single male salmon can mate with several females, selectively harvesting male salmon can increase a fishery’s maximum sustainable harvest. We suggest such selective harvesting of visually distinctive male spawning chum salmon was a common practice, most effectively undertaken at wooden weirs spanning small salmon rivers and streams. We argue that this selective harvesting of males is indicative of an ancient and probably geographically widespread practice for ensuring sustainable salmon populations. The archaeological data presented here confirms earlier ethnographic accounts describing the selective harvest of male salmon.

## Introduction

Indigenous cultures of the Northwest Coast of North America have often been characterized and defined in terms of their subsistence strategies, which involved intensive reliance on abundant marine foods, especially salmon (*Oncorhynchus* spp.), shellfish, and forage fish such as herring (*Clupea pallasii*)^1–3^. Tracing the origins and trajectories of the technological, economic, and cultural adaptations to this natural superabundance of marine resources has been a major focus of regional archaeological research^4–9^. More recently, archaeological, ethnographic, and experimental research is revealing the ways in which the Indigenous peoples of the Pacific Northwest Coast sustained and enhanced desired resources through actively modifying land- and seascapes and selectively harvesting animals and plants^10–19^. Similarly, it has become increasingly clear that Indigenous peoples across the entire coast observed teachings and protocols relating to selective and sustainable harvesting^16–20^. These linked practices were the basis for important technological achievements, complex social and ceremonial alliances, settlement, and territoriality.

**Figure 1:**
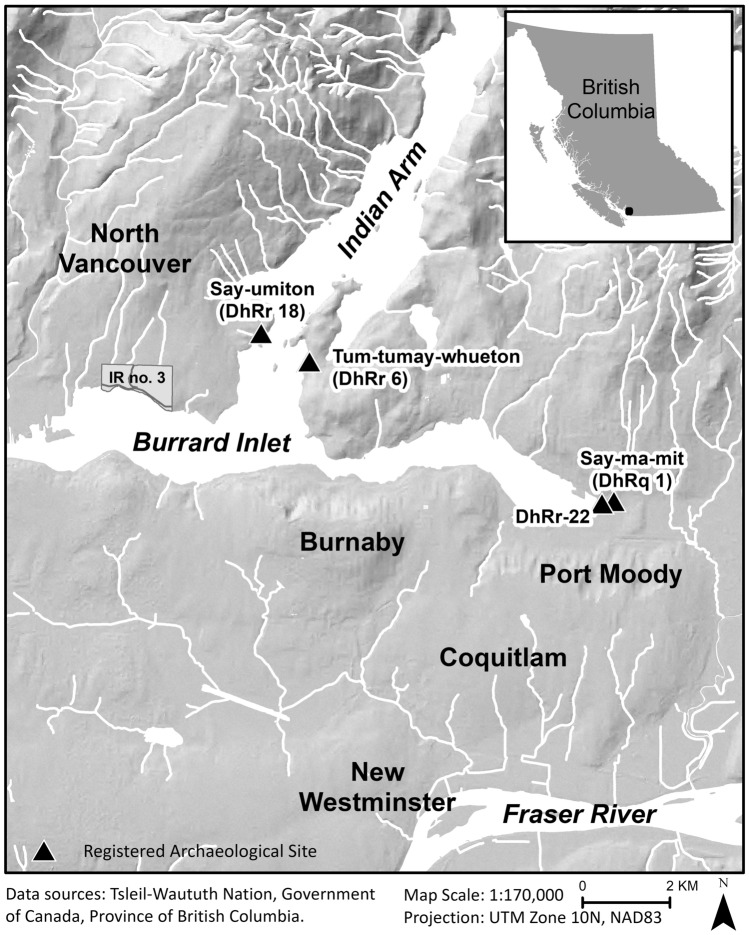
Burrard Inlet and the location of sampled archaeological sites. Figure created in ESRI ArcGIS version 10.8.1 (https://www.esri.com).

In light of this shift in perspective, we examine archaeological evidence for sex-selective harvesting of chum salmon (*Oncorhynchus keta*) at four ancestral Coast Salish settlements located in Burrard Inlet, on the south coast of British Columbia, Canada (Fig. 1). We utilize new palaeogenetic analytical techniques that can identify the sex of archaeological salmonid samples by screening for the presence of the Y-chromosome with PCR assays^21,22^. The significant bias towards male chum salmon observed at two of the sampled sites suggests these communities had sex-selective fisheries. We suggest that this selective harvesting of males is an expression of an ancient and geographically widespread practice for ensuring sustainable salmon populations. This interpretation is strongly supported by traditional knowledge and ethnographic records but has heretofore been impossible to demonstrate archaeologically.

## Background

**Figure 2:**
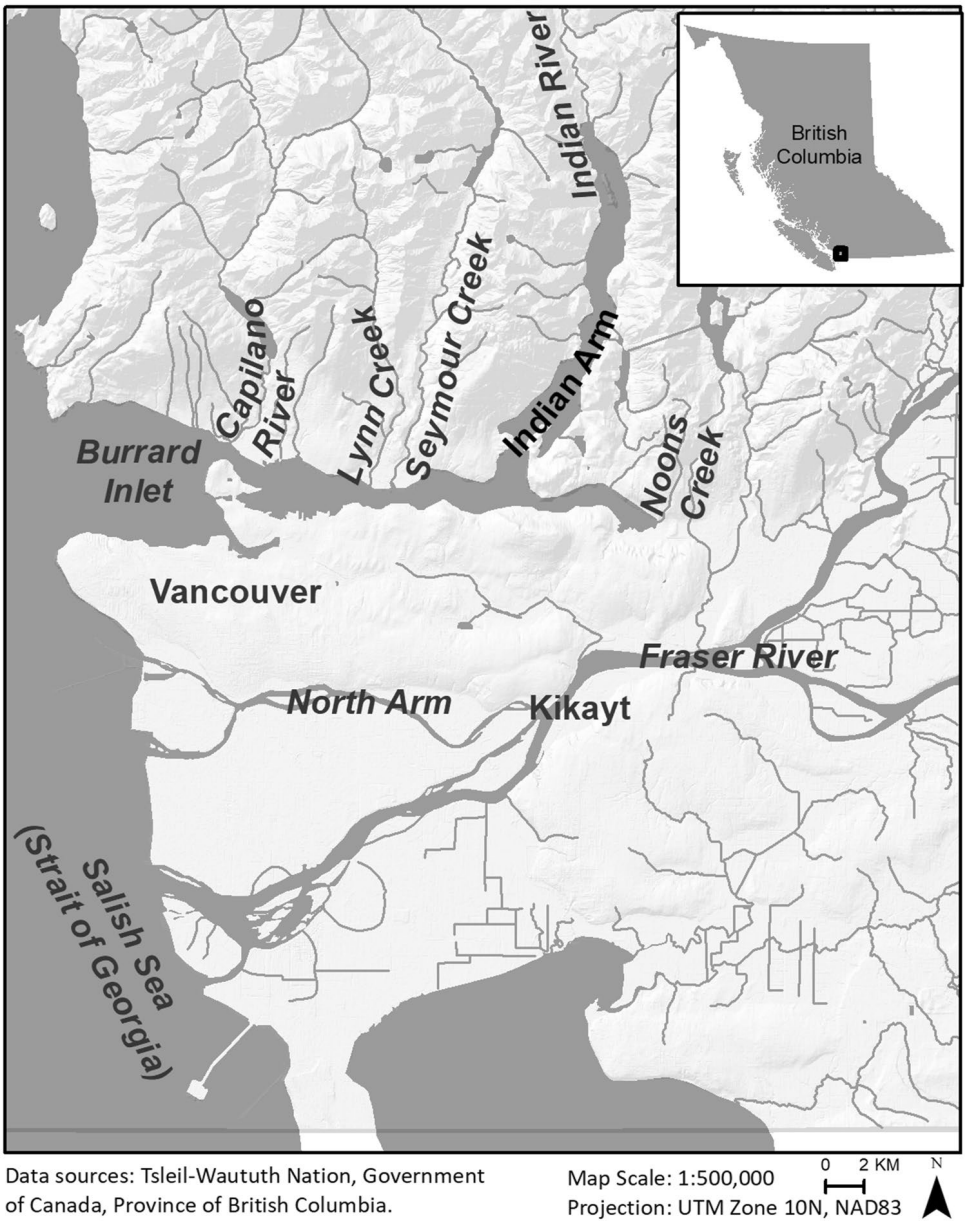
Regional study area and major salmon rivers. Figure created in ESRI ArcGIS version 10.8.1 (https://www.esri.com).

Burrard Inlet (*Tsleil-Waut*) is a narrow inlet on the eastern shore of the Salish Sea that is located immediately north of the present-day city of Vancouver and the Fraser River (Fig. 2). Bounded by low hills to the south and steep mountains to the north, the Inlet is both a geographically and culturally distinct area. The Tsleil-Waututh are a Central Coast Salish group who traditionally spoke a dialect of *Hunq’imnum* and have lived in Burrard Inlet for many thousands of years at least^23–25^. The ancestors of the Tsleil-Waututh people, who live in eastern Burrard Inlet today, had access to a range of salmonids from the salt waters of the Inlet, as well as the many different runs in the various streams and rivers that drain into it, most notably the Capilano, Seymour, and Indian rivers^26,27^. In addition, the Fraser River, which supports the largest salmon runs in North America, was located just 8 km south of Burrard Inlet, and accessible by overland trails or canoe, in which hundreds of salmon could be transported^28^ (Fig. 2).

### Pre-contact fisheries in Burrard Inlet

The pre-contact occupation of eastern Burrard Inlet is primarily known from a number of large shell middens representing the remains of former settlements dating from ca. 3000–200 BP (ca. 1200 BCE–CE 1800)^29–32^. Radiocarbon dates compiled from many of these settlements demonstrate a relatively large population occupied the Inlet continuously from ca. 2300 BP (or ca. 400 BCE)^32^, until catastrophic population losses in the CE 1780s from a smallpox epidemic^24,33^. Analyses of faunal remains and isotopic studies of human remains obtained from excavations at these settlements have identified a diet focussed on marine resources, especially salmon, forage fish, and shellfish^34–38^. Regional archaeological research has identified intensive use of stored salmon beginning around 1500 BCE^9^. It is anticipated that salmon conservation measures, such as selective harvesting, would have developed sometime after this period.

Recent ancient DNA (aDNA) analyses of salmonid remains from these settlements dating from ca. 2300–1000 BP (ca. 400 BCE–CE 1200) has identified a salmon fishery focussed on chum salmon at all sampled sites and components within sites [^38^; Supplementary Table 1]. The overwhelming majority (> 90%) of salmon remains identified through aDNA analysis as chum and pink salmon (*Oncorhynchus gorbuscha*) would have likely been locally harvested within Burrard Inlet, and especially at the rivers emptying into it^38^. In addition to the above-mentioned species, coho (*Oncorhynchus kisutch*), sockeye (*Oncorhynchus nerka)* and Chinook salmon (*Oncorhynchus tshawytscha*) were also possibly harvested within Burrard Inlet, and likely the lower Fraser River, but were recovered in low quantities. Thus, our study focuses on the selective fishing of chum salmon, the dominant salmon species in all Burrard Inlet archaeological assemblages.

After the Fraser River, the Indian River had the largest runs and was the most important fall fishery for Tsleil-Waututh people^23,24,26^ (Fig. 2). The social and economic significance of the Indian River fishery is evident from ancient narratives and from the fact that one of the reserves set aside for the Tsleil-Waututh community in the late nineteenth century was a fishing station near the river's mouth^39^. The majority of the chum harvested by the Tsleil-Waututh in the past century were obtained at the Indian River and we expect that this was the same in the more distant past as there is no evidence for major disruptions (e.g., major landslide) to the river’s salmon populations prior to contact^38^.

Tsleil-Waututh oral histories describe the significance of Indian River salmon and the close relationship between them and Tsleil-Waututh ancestors. One oral history set in ancient times describes how a powerful ancestral Tsleil-Waututh hero overcame a two-headed sea-serpent (Say Nuth Kway)—one head representing disease and the other famine—in Indian Arm that was causing starvation by blocking access to the Indian River^23,40–42^. The implication is that the salmon from Indian River were so important to the Tsleil-Waututh community that without them, there was famine, disease, and death. Another story, set around the time of First Contact (CE 1792), describes how a great Tsleil-Waututh chief punished two boys that were mistreating salmon from the Indian River by making the salmon disappear until the boys learned to demonstrate the proper respect towards salmon^23,43^. These oral histories reflect the importance of salmon to past subsistence, underscore the moral obligations to them, and demonstrate the active role of Tsleil-Waututh ancestors in their continuance.

### Sex-selective salmon harvesting

Chum salmon were one of the most important winter staple foods for the Tsleil-Waututh and other Coast Salish people for several reasons: they return to spawn in the late fall, are abundant in many streams and rivers, are the largest salmon species in the Indian River and the second largest of the seven salmon species found locally, and, due to their relatively low fat content, can be readily smoked and/or dried to last through the winter^44,45^. Selective management of chum populations would ensure resilient and sustainable harvests over many generations, and this stable local resource base would similarly ensure the continuance of local Tsleil-Waututh communities. Experimental evidence and ecological modelling indicate that while the sex ratios of spawning salmon are close to 1:1, a single male will fertilize the eggs of many females, meaning a considerable portion of males can be harvested prior to mating, while maintaining the same egg fertilization rates^46,47^. In the case of sockeye salmon, one experimental study found that a 1:15 male to female ratio (meaning only one male sockeye spawning with 15 females) decreased egg fertilization rates by less than 5%^46^. This means that under normal spawning conditions there is a natural surplus of male salmon and that selective harvesting of male salmon increases a fishery’s maximum sustainable yield.

**Figure 3:**
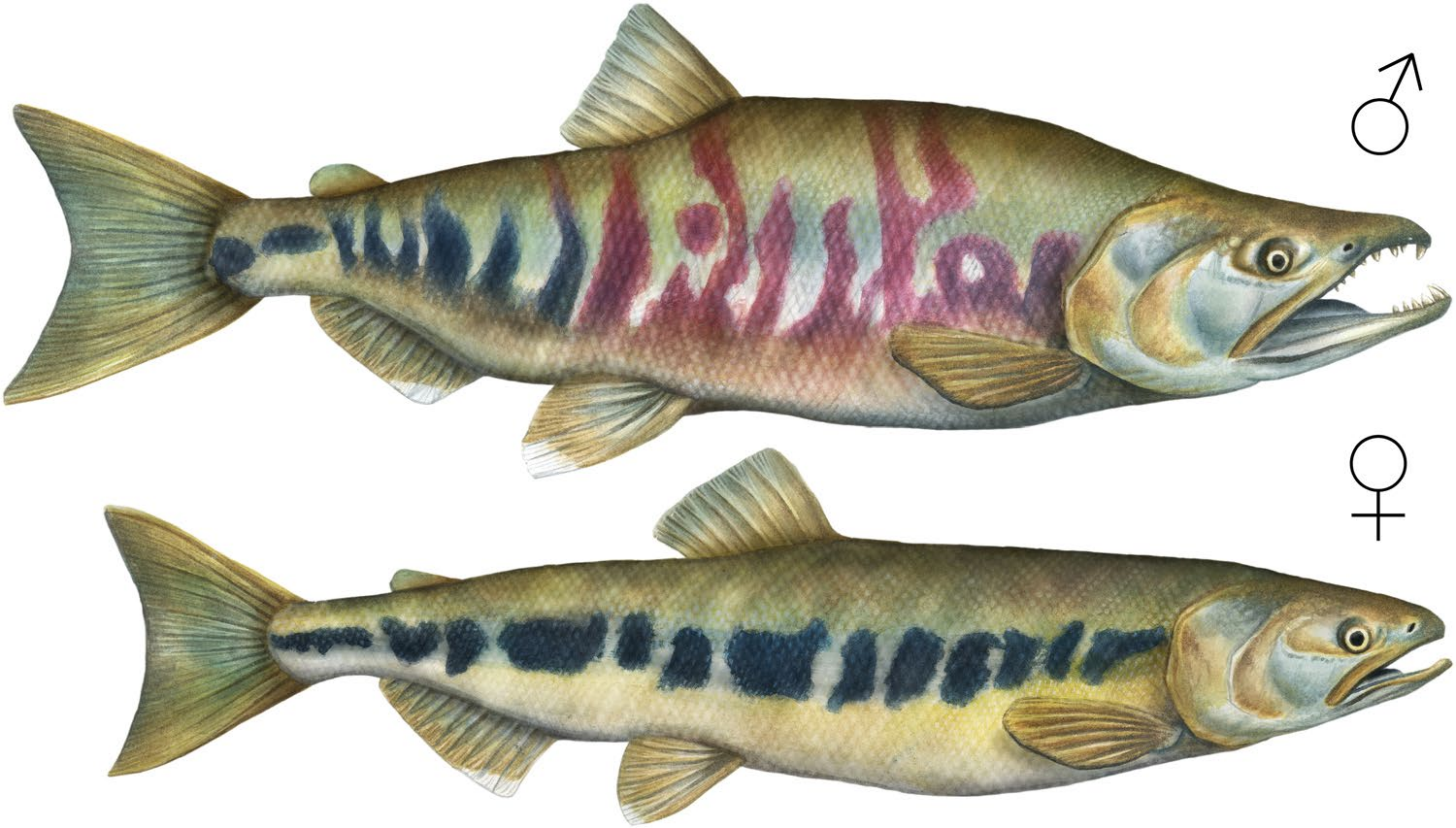
Spawning male (top) and female (bottom) chum salmon (original artwork by Dorian Noël 2020).

As chum salmon approach spawning age, the sexes become easily distinguishable by sight, making it possible to efficiently harvest individuals of a particular sex^48^. Chum males are longer and heavier than females, have large canine teeth, a pronounced hooked jaw (kype), and a mottled red pattern (the “calico nuptial coloration”) on the front portion of their bodies, while the females are smaller and have a distinctive black stripe along their side^48^ (https://www.fisheries.noaa.gov/species/chum-salmon) (Fig. 3). Males also have much more pronounced “humps” on their back, making them far more visible than females in shallow water. This visual difference between male and female chum is well-known to contemporary Tsleil-Waututh fishermen, and also features in Coast Salish oral histories. For example, when Swaneset (a Katzie Coast Salish cultural hero) arrived at the village of the chum (dog) salmon people he saw “that some of the villagers wore red-striped blankets, others black-striped” [^49^; see also ^50^]. Swaneset’s sockeye salmon wife convinced all the salmon people to travel up the Fraser River every year^49^. First, the sockeye salmon people returned, then “when the inhabitants of the Humpback salmon and Dog Salmon villages saw their masters pass, they decided to follow”^49^. This story is set in ancient/mythic times, when many animals and fish were still in the form of people.

Mirroring the above oral history, chum salmon are the last of the local salmon species to spawn each year with spawning occurring in the late fall. Male chum salmon arrive at spawning streams earlier than females, but the overall ratio of males to females approaches 1:1 during the peak of the spawning season^48^. Thus, the sex-ratio of a non-selective chum fishery should be close to 1:1, while a male-selective chum fishery would display a ratio significantly biased towards males.

Ethnographic records indicate that many Coast Salish peoples as well as many other Northwest Coast Indigenous peoples preferentially harvested male salmon^5,51–56^. Conversely, in the Pacific Northwest’s Interior Plateau, female salmon were incidentally preferentially harvested by Interior Salish St'at'imc fishers dip-netting in eddies (not a terminal fishery) along the Mid-Fraser River^57^. Selective fishing was traditionally achieved in several ways. In the Coast Salish area, fish weirs and traps were used by the Cowichan and Sta’ailes^51,52^ to target males. Weirs and traps pen fish alive behind a barrier, which allowed fishers to release female fish and harvest males with nets, baskets, spears, and/or clubs^51,52^. Nighttime spearfishing by torchlight in clear waters was also an effective selective fishing strategy^5^. In the case of the northern Coast Salish Tla’amin people, male salmon were targeted seasonally, with females being avoided only during the first half of the run^5^. Outside of the Coast Salish area, male selective harvesting is best documented among the Tlingit, who modified salmon streams and constructed weirs and traps to facilitate selective spearing and gaffing^47^. Such streamscaping often entailed the removal of rocks from pools in order to expose light coloured substrates, which provided contrast that made identifying male salmon easier^47^. It is important to note that traditional Coast Salish fish weir technology, if used inappropriately, had the potential to destroy local salmon runs by impeding access to their spawning grounds. For this reason, only chiefs, weir specialists and shamans who carried the specific traditional knowledge regarding particular weir sites were charged with the proper construction and utilization of fish weirs for their communities^20^. Terminal weir fisheries were sustainable for many centuries in the past only because of the appropriate cultural teachings that were tied to their use.

The conservation benefits of male-selective salmon fisheries were well recognized by the Coast Salish and other Indigenous peoples, particularly those that lived in villages adjacent to spawning channels and other terminal fisheries. For instance, at the densely occupied confluence of the Harrison and Chehalis Rivers in the eastern Fraser Valley, Elder Dana Charlie described how Sts’ailes people targeted male chum salmon to the greatest extent possible in order to ensure salmon would “come back year after year”^52^. This sex-based selection was one of the most important teachings^52^. Similarly, Chief Tom of the Tla’amin stated that the purpose of not harvesting female salmon was “to make more”^5^. Non-Coast Salish Indigenous peoples, such as the Tlingit^54^, also recognized the relationship between the sustainability of salmon fisheries, and male-selective fishing strategies.

In addition to conservation concerns, male salmon were also preferred over females by Coast Salish fishers on account of their larger size. As Dana Charlie notes:

The other reason [for targeting male salmon] is that males have more meat. You lose 4–5 pounds from a 10 pound (female) salmon to bones and the reproductive system. On a 10 pound male, you only lose about 1 pound of that. There’s not much cavity inside the male like there is in the female. I won’t clean a female for smoking, there’s just not enough flesh there. Kind of a waste of time, for me it is^52^.

Further afield, some Ahtna and Tlingit individuals express a similar preference for male salmon due to their larger size^54,55^. It is this larger size that made male chum salmon more vulnerable to other predators as well. Tlingit fishers also note that on occasion male-biased salmon harvests occurred due to stochastic variations in the sex composition of spawning runs rather than deliberate selection^54^.

In summary, archaeological evidence indicates that the ancestral Coast Salish inhabitants of eastern Burrard Inlet had a marine based diet focussed on local resources, especially chum salmon, forage fish and shellfish^30,38^. Drawing on archaeological data and oral history, we emphasized the connections between long-term human occupation at spawning channels, sex-selective salmon fisheries, and the moral responsibility to care for salmon. We also demonstrate how these traditional teachings are aligned with scientific studies showing how male-biased harvesting contributes to a sustainable salmon fishery. To assess if sex-selective salmon fishing similar to that described in the ethnographic record was practiced by the Coast Salish prior to contact, we used aDNA analysis to investigate the sex-selectivity of several pre-contact salmon fisheries in the Burrard Inlet.

## Materials and methods

Archaeological salmon bone was selected for aDNA analysis from previously excavated materials from four pre-contact settlements in Burrard Inlet (Fig. 1). A fifth site, represented by a single sample, is excluded from this discussion [Supplementary Table 1]. The samples were selected from contexts providing geographic and temporal coverage of eastern Burrard Inlet sites and date from about 2300–1000 BP (ca. 400 BCE–CE 1200) [^31,38^, Supplementary Table 1]. Samples of salmon bone were selected from 17 distinct radiocarbon dated contexts within these four settlements. Whole salmon vertebrae were initially selected for sampling based on their state of preservation following Cannon and Yang^8^. In contexts containing relatively few salmon bones (i.e., less than five), all available samples were selected. When more than five salmon vertebrae (occasionally many thousands of vertebrae) were present, a random sampling procedure was employed to avoid sample bias^8^. It is exceedingly unlikely that the samples of salmon vertebrae derived from each of these sites were drawn from the same individual salmon, as our sampling procedure randomly selected salmon vertebrae from several stratigraphic contexts from each archaeological site. In total, 116 salmon vertebrae were selected for aDNA analysis with the goal of identifying the sex of each sample. Species-level identifications were previously assigned to these samples through aDNA analysis by Morin et al.^38^.

### Decontamination and DNA extraction

Decontamination, DNA extraction, and PCR setup were all conducted in a dedicated ancient DNA laboratory in the Department of Archaeology, Simon Fraser University (Burnaby, BC, CA), that is physically separated from the post-PCR laboratory. Rigorous contamination control protocols were followed throughout the analysis^58^. All the samples were decontaminated prior to DNA extraction through a combination of bleach washes and UV irradiation^59^. DNA was extracted from each sample following a modified silica-spin column method^60,61^. To assess the replicability of our results, DNA extraction was repeated for 11 of the samples (BIS86, BIS87, BIS89, BIS90, BIS91, BIS92, BIS95, B1S101, BIS104, BIS107, BIS109). To detect instances of contamination, blank extraction controls were processed in tandem with the samples and subjected to amplification. For the technical details regarding the decontamination and DNA extraction procedures employed, please refer to Morin et al.^38^, which analyzed the same set of samples.

### Sex identification

Sex identifications were assigned to the analyzed salmonid remains using two PCR assays (*clock1a*/*sdY* and D-loop/*sdY*) developed by Royle et al.^21^. Both assays use two sets of primers to co-amplify a fragment of the Pacific salmonid Y-linked master sex-determining gene (*sdY*)^62–64^ and an internal positive control (IPC) consisting of a fragment of the nuclear *clock1a* gene (*clock1*a/*sdY* assay) or mitochondrial D-loop (D-loop/*sdY* assay) (Table 1). PCR amplifications were performed on a Mastercycler Gradient or Personal thermal cycler (Eppendorf, Mississauga, ON, CA) in a 30 μL reaction volume. The reaction volume for the PCR assays contained 1.5 × PCR Gold Buffer (Applied Biosystems, Austin, TX, USA), 2 mM MgCl_2_, 0.2 mM dNTP, 0.45 μM (*clock1*a/*sdY* assay) or 0.6 μM (D-loop/*sdY* assay) of each *sdY* primer (Table 1), 0.3 μM of each *clock1a* primer (*clock1a*/*sdY* assay) or 0.1 μM of each D-loop primer (D-loop/*sdY* assay) (Table 1), BSA (1 mg/mL), 3 μL aDNA sample, and 0.75 U AmpliTaq Gold (Applied Biosystems, Austin, TX, USA). The thermal conditions for the PCR assays consisted of an initial denaturation step at 95 °C for 12 min followed by 60 cycles at 95 °C for 30 s (denaturation), 54 °C for 30 s (annealing), and 70 °C for 40 s (extension), and a final extension step at 72 °C for 7 min. Negative PCR controls were included in each PCR run in order to monitor for contamination.

**Table 1:** Primers used in the PCR sex identification assays.

Primer	Locus	Sequence (5′–3′)	Amplicon size (bp)	References
*Clk1*a-F50^1^	*clock1a*	TAGCCATGTCTGTGTGTTTACTTGC	108	^21^
*Clk*1a-R60		GCAGCCAGCTAATTKGATTTG		^21^
Smc7 (F)	D-loop	AACCCCTAAACCAGGAAGTCTCAA	249	^65^
Smc8 (R)		AACCCCTAAACCAGGAAGTCTCAA		^65^
*sdY*-F19	*sdY*	CCCAACACCCTTCCTATCTCC	95	^21^
*sdY*-R20		CCTTCCTCCCTAGAGCTTAAAAC		^21^

^1^F and R denote forward and reverse primers, respectively.

Following amplification, 3–5 μL of PCR product from each sample was pre-stained with SYBR Green I (Invitrogen, Eugene, OR, USA), electrophoresed on a 2% or 3% agarose gel, and visualized with a Dark Reader transilluminator (Clare Chemical Research, Dolores, CO, USA). Sex identities were assigned to the samples based on a visual examination of the resulting electrophoresis gels. In brief, a sample was identified as male if *sdY* was amplified with one of the assays, while a female identity was assigned to a sample if the IPCs, but not *sdY*, were amplified with both assays. No sex identity was assigned if these criteria were not met. Statistical analyses of the sex identification results were performed in R version 3.6.2^66^.

## Results and discussion

**Figure 4:**
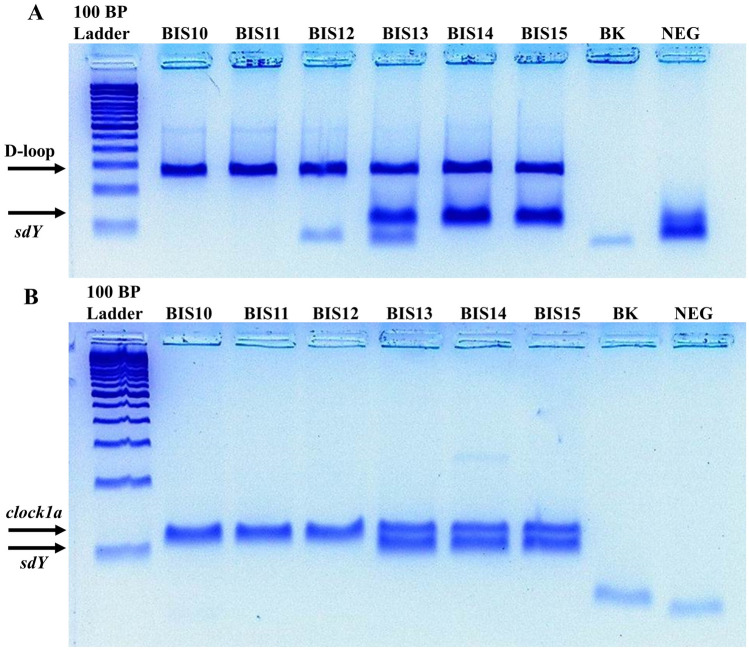
Negative images of electrophoresis gels showing the results of the (**A**) D-loop/*sdY* and (**B**) *clock1a*/s*dY* PCR sex identification assays for six of the analyzed Pacific salmon (*Oncorhynchus* spp.) samples (BIS#). The approximate positions of the internal positive control (D-loop and *clock1a*) and *sdY* amplicons are indicated by the labelled arrows. BK denotes the blank extraction control processed alongside the samples. NEG denotes negative PCR controls. The 100 bp ladder is from Invitrogen (Vilnius, LT). PCR products were pre-stained with SYBR Green I (Invitrogen, Eugene, OR, USA). Unprocessed images of the electrophoresis gels are presented in Supplementary Fig. 1 and Supplementary Fig. 2.

Of the 116 samples examined in this study, 96 samples were successfully assigned a sex identity using the criteria outlined above. Of these 96 samples, 87 samples yielded consistent results across both assays. An additional 9 samples were identified as males as *sdY* was successfully amplified with one of the assays, but was not amplified with the other assay, likely as a result of allelic dropout. The sex identities assigned to the 11 samples that underwent repeat DNA extraction were consistent across both extractions. No DNA was amplified with the *clock1a*/*sdY* or D-loop/*sdY* assay from any of the blank extraction or negative PCR controls. Figure 4 presents exemplar gels showing the results of the two sex identification assays for a subset of the analyzed samples. Detailed amplification and sex identification results for each of the analyzed samples are presented in Supplementary Table 1.

To increase our sample size, we included 9 chum and 2 pink salmon samples previously sexed by Royle et al.^21^ using the same methodology and derived from the same dated archaeological sites as the larger body of samples in our statistical analyses. We excluded a single sample collected from an additional site (DhRr 20) that was analyzed here from the succeeding statistical analyses and discussion due to the small sample size (n = 1). This single sample from DhRr 20 was a female chum. Table 2 summarizes the sex identifications results across all salmonid species.

**Table 2:** Summary of sexed salmon by archaeological site irrespective of species. Statistically significant results at a α = 0.05 level of significance are in bold font and marked with an asterisk.

Site	Male salmon	Female salmon	No sex ID assigned	Sex ratio	Binomial exact test (two-tailed) *P* value
DhRq 1	8	9	3	1–1.125	1.0
DhRr 6	33	17	17	1–.52	0.03*
DhRr 18	17	14	0	1–1	0.72
DhRr 22	7	1	0	1–.143	0.07

When these results are summarized by archaeological site irrespective of species, it is clear that among the four well-sampled sites two (*Say-umiton*/DhRr 18 and *Say-ma-mit*/DhRq 1) have sex ratios very close to the 1:1 (Table 2) ratio expected for a non-sex selective salmon harvest, while another two (*Tum-tumay-wheuton*/DhRr 6 and DhRr 22) have sex ratios that are markedly biased towards males (Table 2). A binomial exact test (two-tailed) indicates the assemblage of sexed salmon remains from *Tum-tumay-wheuton*/DhRr 6 is significantly male-biased at a *α* = 0.05 level of significance (*p* = 0.033). These results indicate it is very unlikely that this sample was drawn from a population of salmon remains that has an approximately 1:1 male to female sex ratio.

**Figure 5:**
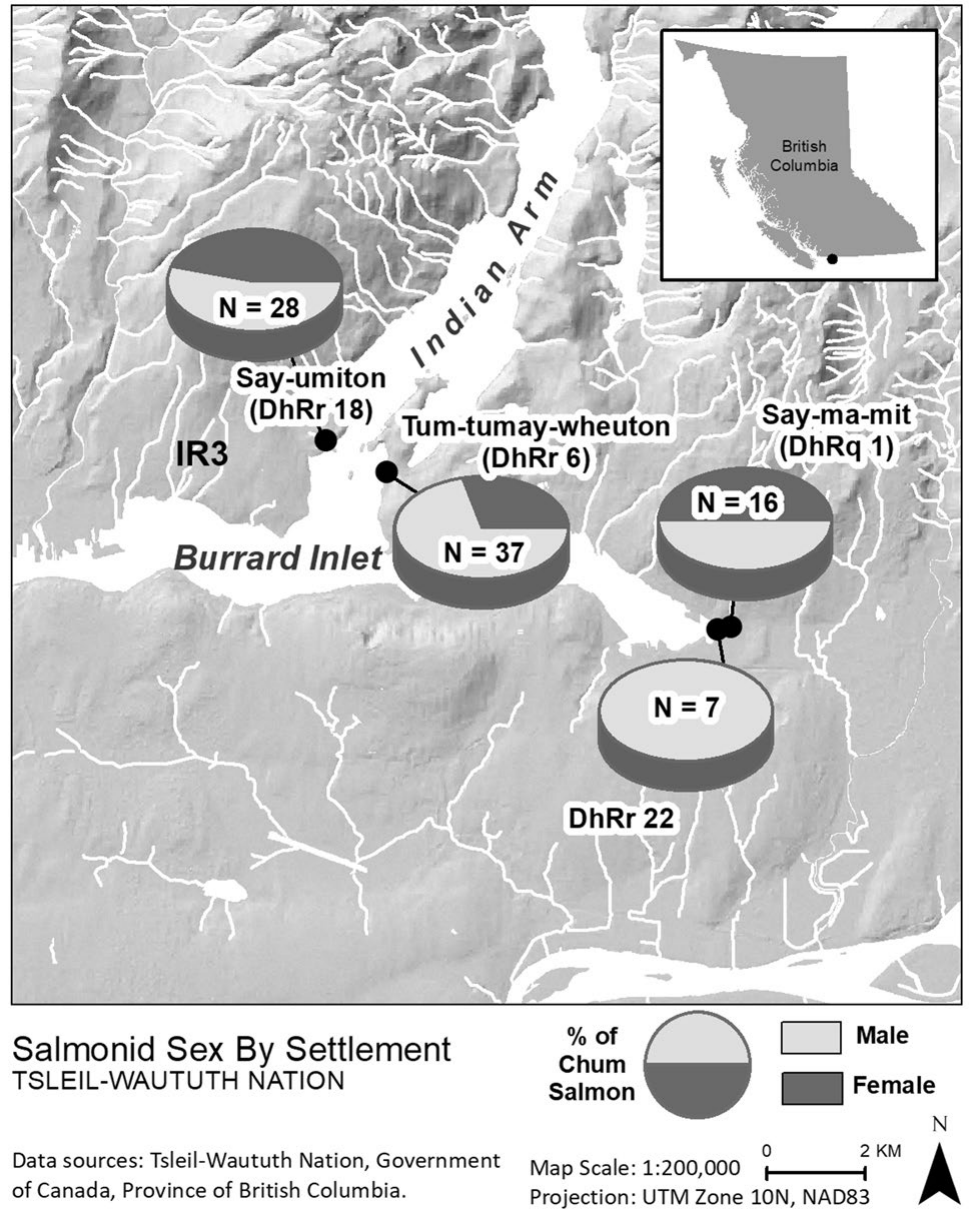
Proportion of male and female chum salmon at the sampled archaeological sites. Figure created in ESRI ArcGIS version 10.8.1 (https://www.esri.com).

Upon further inspection of these data, it was clear that bias towards males was especially pronounced among the chum salmon remains, which is by far the most common species in these assemblages^38^ (Table 3, Fig. 5). A binomial exact test (two-tailed) indicates the sexed chum salmon assemblages from *Tum-tumay-wheuton*/DhRr 6 (*p* = 0.02), and DhRr 22 (*p* = 0.02) are both significantly male-biased at a *α* = 0.05 level of significance (Table 3). Again, these results indicate that it is very unlikely that these chum remains were drawn from a population with a 1:1 sex ratio. This is strong evidence for preferential harvesting of male chum salmon by the inhabitants of *Tum-tumay-wheuton*/DhRr 6 and DhRr 22 prior to contact. We interpret this bias towards male chum as evidence for the pre-contact use of sex-selective fishing as a resource management strategy.

**Table 3:** Summary of sexed chum salmon by archaeological site. Statistically significant results at a α = 0.05 level of significance are in bold font and marked with an asterisk.

Site	Male chum	Female chum	Sex ratio	Binomial exact test (Two-tailed) *P*-value
DhRq 1	8	8	1–1	1.0
DhRr 6	26	11	1–.42	0.02*
DhRr 18	15	13	1–0.87	0.85
DhRr 22	7	0	1–.14	0.02*

Theoretically, the sex ratios we observed could have been the product of PCR issues rather than cultural practices. As a result of DNA degradation and inhibition, allelic dropout of sex-linked markers can occur, resulting in erroneous sex identifications being assigned to samples with PCR-based methods such as the one employed here^67,68^. When it occurs and the X-chromosome markers (or in this case a proxy for the X-Chromosome) still amplify, Y-chromosome dropout can result in the misidentification of male samples as female. If this did occur, it would only act to obscure the actual number of male samples represented in assemblages, thus enhancing the argument of preferential selection of males over females. In addition to allelic dropout, contamination may also result in erroneous sex identifications. However, the failure to amplify DNA from any of the blank extraction or negative PCR controls, suggests systematic contamination did not occur^69^. The successful replication of the sex identities assigned to most samples through two independent PCR assays and repeat DNA extractions also supports the authenticity of the aDNA data.

The most parsimonious explanation for the observed bias towards male chum at *Tum-tumay-wheuton*/DhRr 6 and DhRr 22 is that it is the result of pre-contact sex-selective harvesting as part of a resource management strategy similar to those ethnographically described in the Pacific Northwest. As described above, in recent times chum salmon were caught by Coast Salish people in the late fall as they began to ascend the salmon streams of Burrard Inlet, especially the Indian River. Chum salmon are weak jumpers, and in pre-contact times, they were very likely harvested from rivers and creeks impounded with weirs and traps. As documented in the ethnographic record, trapped male chum could have been retrieved from traps after easily being visually identified based on their larger size, marked red pattern, and large canines, while many of the females were free to ascend and spawn upstream. Such a selective harvesting regime would act to increase the chum harvest without any risk of depressing future stocks, but we cannot be certain this was the goal of the selective fishery. The consistently high abundance of chum salmon remains at sites in the Burrard Inlet throughout the past 2000 years^38^ indicate sex-selective fishing and other management strategies were indeed successful.

While all of our sexed salmon samples date from about 2300–1000 BP (ca. 400 BCE–CE 1200), the chum salmon assemblages showing the strong male bias span a narrower range of time. The sexed chum salmon samples from *Tum-tumay-wheuton*/DhRr 6 were recovered from strata dating from about 1710–1070 BP (CE 200–1160) (Supplementary Table 1). The sexed chum recovered from DhRr 22 date to about 1760 BP (CE 180–380). This pattern of sex-selective chum fisheries in Burrard Inlet is therefore most clear from about 1760 BP to about 1070 BP (CE 180–1160), but we expect that additional samples would expand this date range.

It is important to note, however, that not all sites in Burrard Inlet display this bias towards male chum salmon. Our sampled chum from *Say-ma-mit/*DhRq 1, dating to about 2300–1600 BP (400 BCE – CE 550), displayed no evidence of bias towards male chum. This contrasts with the contemporaneous DhRr 22, where, as noted above, a strong male bias was observed among chum. These two contemporaneous sites are located about 200 m apart along the shoreline of Port Moody Inlet, with DhRl 22 being immediately adjacent to Noon’s Creek. This marked difference in chum harvesting strategies between contemporaneous and neighbouring sites requires explanation.

One possible explanation for this difference may be as simple as differences in methods of capture, with the inhabitants of DhRr 22 taking more active responsibility for the creek that ran past their village (perhaps using weirs and traps to selectively capture male chum), and the inhabitants of *Say-ma-mit*/DhRq 1 using nets to capture male and female chum indiscriminately. A second possible explanation is that the inhabitants of DhRr 22 had the rights to harvest chum at a major weir, such as one on the Indian River where the stocks were closely managed, while the inhabitants of *Say-ma-mit*/DhRq 1 did not have such rights, and instead harvested chum indiscriminately from several streams draining into Port Moody, including Noon’s Creek. Along similar lines, we suggest that the differences between the nearby villages of *Tum-tumay-wheuton*/DhRr 6 and *Say-umiton*/DhRr 18, likely have to do with differential access to the most productive salmon-streams, such as the Indian River where selective harvesting could be more effectively undertaken [Supplementary Table 1].

A second possible explanation for the bias towards male chum at two sites and the lack of such a bias at two other sites could be related to transport costs. For example, perhaps those families who harvested chum at some distance from their primary settlements were more inclined towards harvesting large male chum rather than smaller females. These groups would then, at the end of the chum season, return to their primary villages with canoe loads of dried/smoked chum, especially the larger males. Conversely, families who harvested chum from streams and creeks located very close to their primary settlements would return with these fish every day. In this case, bulk transport of large quantities of preserved fish would not be a primary consideration. With our current knowledge of these sites, we are presently unable to evaluate these possible explanations for the presence or absence of sex-selective fisheries at specific sites in Burrard Inlet.

On the Northwest Coast, similar variation in marine resource management strategies has been observed in other locales. At *Ts’ishaa* on Vancouver Island (ca. 1800–250 BP), McKechnie^70^ observed inter-household differences in fisheries management strategies, specifically the size-selectivity of their rockfish fisheries. A range of studies has also highlighted how the species targeted by Indigenous fisheries in Northwest Coast varied between sites [e.g.,^71–74^]. This reflects the fact that Indigenous traditional ecological knowledge is not universal but rooted in specific places, the cumulative multi-generational product of many individuals’ entwined lived experiences of the ecological and cultural realties of a particular time and locale^75^.

With regards to the other salmon species, there are good reasons why such a selective harvest in our study area would have been difficult or impossible to undertake. Sockeye salmon were likely harvested in large nets set between canoes in the murky Fraser River, or in the marine waters off of Point Roberts^5^. Each successive set would entrap scores or hundreds of fish^76^. These loaded nets would be hauled ashore where the struggling fish quickly die, males and females alike, in roughly equal proportions. Also, if salmon were harvested from saltwater while trolling from canoe (most likely pink and coho in Burrard Inlet), fishermen would have had roughly equal chances of landing males and females. Moreover, it would have been difficult to determine the sex of the landed fish as ocean-phase salmon do not exhibit marked sexual dimorphism. For these reasons, the specific characteristics of chum salmon, and the methods for harvesting chum salmon makes it more suitable for a selective harvest compared to other salmon species. This practice of selectively harvesting male chum should be considered an aspect of the pre-contact traditional ecological knowledge used by ancestral Coast Salish people to increase local sustainable harvests of their winter staple.

This evidence for the past selective harvesting of male chum fits within a broader pattern of Indigenous resource enhancement and management strategies in the Pacific Northwest. Such resource enhancement and management strategies include: the use of clam gardens^10,13,77^, estuarine gardens^78^, wapato gardens^79^, prescribed burning to enhance plant growth^80^, and the selective harvesting and propagation of plants^81–83^. Recently, researchers have argued for the “cultivation” of salmon via habitat enhancement, transplanting, and stewardship principles^17^. The male-selective chum fisheries we identified in the Burrard Inlet at *Tum-tumay-wheuton*/DhRr 6 and DhRr 22 should be considered a central principle in sustainable and resilient salmon trusteeship^54^. Globally, other examples of pre-contact/pre-industrial selective fisheries have been identified [e.g.,^84,85^] and we expect that further research will reveal that such selective fisheries were once widespread, and partly responsible for the long-term resilience of salmon throughout so many densely occupied waterways.

## Conclusion

The diversity of pre-contact Indigenous resource management techniques in the Pacific Northwest are becoming increasingly apparent to archaeologists^10,13,77–83^. To assess the possibility of sex-selective salmon fishing in the region, we used new DNA-based methods to identify the sex of archaeological salmon samples from a number of sites around Burrard Inlet dating from about 2300–1000 BP (ca. 400 BCE–CE 1200). A statistically significant bias towards male chum salmon was identified at two sites—*Tum-tumay-wheuton*/DhRr 6 and DhRr 22—dating to ca. 1710–1070 BP (ca. CE 90–1160) and 1760 BP (ca. CE 180–380), respectively. We interpret this bias towards male chum as a product of a sex-selective fishery preferentially targeting the surplus sex, males, with conservation goals in mind. Evidence for this selective salmon fishery appears approximately 1500 years after the earliest local evidence for heavy reliance on stored salmon^9^.

The pronounced differences between male and female chum are and no doubt were apparent to Indigenous fishers in Burrard Inlet, and the ease with which they can be captured using weirs make chum suitable for sex-selective fishing. Guided by vested interests and informed by countless generations of close observation of salmon spawning behaviours, and monitoring of annual salmon returns, the pre-contact Coast Salish inhabitants of Burrard Inlet were able to make stewardship decisions regarding chum salmon. These new archaeological data confirm earlier ethnographic descriptions of the preferential harvesting of male salmon by the Indigenous peoples of the Pacific Northwest^5,51–56^. Given our results, we anticipate that with appropriate sampling, other examples of sex-selective salmon fisheries will be identified in the Northwest Coast.

Understanding the range of strategies Northwest Coast Indigenous peoples used to manage salmon stocks also has important implications for present-day Pacific salmon stocks in the region, many of which are imperiled or extinct^86–90^. Indigenous terminal fisheries using weir technology could have destroyed local salmon runs, but there is no evidence that this occurred on the Northwest Coast^16^. Instead, selective terminal fisheries governed by specific cultural teachings and protocols ensured an abundant and reliable resource for many centuries^16,20^. Conversely, in Canada and beyond, past and present government fisheries management approaches informed by resource commodification rather than a culturally rooted strong stewardship ethic have been ineffective at preventing salmon population declines^89,90^. By pairing them with Western conservation approaches on co-equal terms, time-tested Indigenous fisheries management strategies, such as selectively harvesting males, can enhance the sustainability of present-day salmon fisheries as they have done for millennia^16,91–93^.

## Supplementary Information

Supplementary Information.
