# Supplementary material for: Indigenous sex-selective salmon harvesting demonstrates pre-contact marine resource management in Burrard Inlet, British Columbia, Canada
Source: Sci Rep. 2021 Nov 10;11:21160. doi: 10.1038/s41598-021-00154-4 (PMC8581006; doi:10.1038/s41598-021-00154-4)
Supplement: Supplementary file 1 — Supplementary Information. [file 41598_2021_154_MOESM1_ESM.pdf]

Supplementary Information for "Indigenous Sex-Selective Salmon Harvesting Demonstrates Pre-Contact Marine Resource Management in Burrard Inlet, British Columbia, Canada"

Jesse Morin, Thomas C.A. Royle, Hua Zhang, Camilla Speller, Miguel Alcaide, Ryan Morin, Morgan Ritchie, Aubrey Cannon, Michael George, Michelle George, and Dongya Yang

Supplementary Table 1. Provenience information and amplification results for the analyzed salmonid samples. P indicates successfully amplified fragments whereas N indicates fragments that failed to amplify. Samples identified as female are denoted with an F while samples identified as male are denoted with an M.

Lab Code	Site Name	Borden Number	Context	Date Range (uncal BP)	Date Range (2-sigma range cal BCE/CE)	Species ID	Common Name	D-loop/*sdY* Assay		*clock1a*/*sdY* Assay		Consensus Sex ID
								D-Loop	*sdY*	*clock 1a*	*sdY*	
**BIS1**	–	DhRr 20	Sampling unit #13	380 +/- 36 BP	CE 1442-1634	*O. keta*	chum	P	N	P	N	F
**BIS10**	*Tum-tumay-wheuton*	DhRr 6	114-116 N, 3-5W, 40-50 cm	1620-1070 BP	CE 235-1164	*O. keta*	chum	P	N	P	N	F
**BIS100**	*Say-ma-mit*	DhRq 1	10-12s, 35-36w, 60-70 cm	1774 +/-67 BP	CE 86-409	*O. keta*	chum	P	P	P	P	M
**BIS101**	*Say-ma-mit*	DhRq 1	10-12s, 35-36w, 60-70 cm	1774 +/-67 BP	CE 86-409	*O. keta*	chum	P	N	P	N	F
**BIS102**	*Say-ma-mit*	DhRq 1	10-12s, 35-36w, 60-70 cm	1774 +/-67 BP	CE 86-409	*O. keta*	chum	P	N	P	N	F
**BIS103**	*Say-ma-mit*	DhRq 1	10-12s, 35-36w, 60-70 cm	1774 +/-67 BP	CE 86-409	*O. keta*	chum	P	P	P	P	M
**BIS104**	*Say-ma-mit*	DhRq 1	10-12s, 35-36w, 60-70 cm	1774 +/-67 BP	CE 86-409	*O. keta*	chum	P	P	P	P	M
**BIS105**	*Say-ma-mit*	DhRq 1	10-12s, 35-36w, 60-70 cm	1774 +/-67 BP	CE 86-409	—	—	N	N	N	N	—
**BIS106**	*Say-ma-mit*	DhRq 1	10-12s, 35-36w, 60-70 cm	1774 +/-67 BP	CE 86-409	*O. kisutch*	coho	P	N	P	N	F
**BIS107**	*Say-ma-mit*	DhRq 1	15-17s, 32-33w, 30-55 cm	1962-1594 BP	54 BCE to CE 560	*O. keta*	chum	P	P	P	P	M
**BIS108**	*Say-ma-mit*	DhRq 1	15-17s, 32-33w, 30-55 cm	1962-1594 BP	54 BCE to CE 560	*O. keta*	chum	P	N	P	N	F

**BIS109**	*Say-ma-mit*	DhRq 1	15-17s, 32-33w, 30-55 cm	1962-1594 BP	54 BCE to CE 560	*O. keta*	chum	P	P	P	P	M
**BIS11**	*Tum-tumay-wheuton*	DhRr 6	114-116 N, 3-5W, 40-50 cm	1620-1070 BP	CE 235-1164	*O. keta*	chum	P	N	P	N	F
**BIS110**	*Say-ma-mit*	DhRq 1	15-17s, 32-33w, 30-55 cm	1962-1594 BP	54 BC to CE 560	*O. keta*	chum	P	N	P	N	F
**BIS111**	*Say-ma-mit*	DhRq 1	15-17s, 32-33w, 30-55 cm	1962-1594 BP	54 BCE to CE 560	*O. keta*	chum	P	N	P	N	F
**BIS112**	*Say-ma-mit*	DhRq 1	15-17s, 32-33w, 30-55 cm	1962-1594 BP	54 BCE to CE 560	*O. keta*	chum	P	P	P	P	M
**BIS113**	*Say-ma-mit*	DhRq 1	15-17s, 32-33w, 30-55 cm	1962-1594 BP	54 BCE to CE 560	*O. keta*	chum	P	N	P	N	F
**BIS114**	*Say-ma-mit*	DhRq 1	15-17s, 32-33w, 30-55 cm	1962-1594 BP	54 BC to AD 560	*O. keta*	chum	P	N	P	N	F
**BIS115**	*Say-ma-mit*	DhRq 1	15-17s, 32-33w, 30-55 cm	1962-1594 BP	54 BCE to CE 560	*O. keta*	chum	P	P	P	P	M
**BIS116**	*Say-ma-mit*	DhRq 1	15-17s, 32-33w, 30-55 cm	1962-1594 BP	54 BCE to CE 560	*O. keta*	chum	N	N	N	N	—
**BIS12**	*Tum-tumay-wheuton*	DhRr 6	114-116 N, 3-5W, 40-50 cm	1620-1070 BP	CE 235-1164	*O. keta*	chum	P	N	P	N	F
**BIS13**	*Tum-tumay-wheuton*	DhRr 6	114-116 N, 3-5W, 40-50 cm	1620-1070 BP	CE 235-1164	*O. keta*	chum	P	P	P	P	M
**BIS14**	*Tum-tumay-wheuton*	DhRr 6	114-116 N, 3-5W, 40-50 cm	1620-1070 BP	CE 235-1164	*O. gorbuscha*	pink	P	P	P	P	M
**BIS15**	*Tum-tumay-wheuton*	DhRr 6	114-116 N, 3-5W, 40-50 cm	1620-1070 BP	CE 235-1164	*O. gorbuscha*	pink	P	P	P	P	M
**BIS16**	*Tum-tumay-wheuton*	DhRr 6	114-116 N, 3-5W, 40-50 cm	1620-1070 BP	CE 235-1164	*O. keta*	chum	N	N	P	N	—
**BIS17**	*Tum-tumay-wheuton*	DhRr 6	114-116 N, 3-5W, 40-50 cm	1620-1070 BP	CE 235-1164	*O. gorbuscha*	pink	P	N	P	N	F
**BIS18**	*Tum-tumay-wheuton*	DhRr 6	114-116 N, 3-5W, 40-50 cm	1620-1070 BP	CE 235-1164	*O. keta*	chum	N	N	N	N	—
**BIS19**	*Tum-tumay-wheuton*	DhRr 6	114-116 N, 3-5W, 40-50 cm	1620-1070 BP	CE 235-1164	*O. gorbuscha*	pink	P	N	N	N	—
**BIS2**	*Tum-tumay-wheuton*	DhRr 6	Auger 8 level #6	1174 BP	CE 728-971	*O. keta*	chum	P	P	P	P	M
**BIS20**	*Tum-tumay-wheuton*	DhRr 6	114-116 N, 3-5W, 40-50 cm	1620-1070 BP	CE 235-1164	*O. gorbuscha*	pink	P	P	P	P	M

**BIS21**	*Tum-tumay-wheuton*	DhRr 6	114-116 N, 3-5W, 40-50 cm	1620-1070 BP	CE 235-1164	*O. keta*	chum	P	P	P	P	M
**BIS22**	*Tum-tumay-wheuton*	DhRr 6	114-116 N, 3-5W, 40-50 cm	1620-1070 BP	CE 235-1164	*O. keta*	chum	P	N	P	N	F
**BIS23**	*Tum-tumay-wheuton*	DhRr 6	114-116 N, 3-5W, 40-50 cm	1620-1070 BP	CE 235-1164	*O. keta*	chum	P	P	P	P	M
**BIS24**	*Tum-tumay-wheuton*	DhRr 6	114-116 N, 3-5W, 40-50 cm	1620-1070 BP	CE 235-1164	*O. keta*	chum	P	P	P	P	M
**BIS25**	*Tum-tumay-wheuton*	DhRr 6	114-116 N, 3-5W, 40-50 cm	1620-1070 BP	CE 235-1164	*O. keta*	chum	P	P	P	P	M
**BIS26**	*Tum-tumay-wheuton*	DhRr 6	114-116 N, 3-5W, 40-50 cm	1620-1070 BP	CE 235-1164	*O. keta*	chum	P	N	P	N	F
**BIS27**	*Tum-tumay-wheuton*	DhRr 6	114-116 N, 3-5W, 40-50 cm	1620-1070 BP	CE 235-1164	*O. keta*	chum	P	P	P	P	M
**BIS28**	*Tum-tumay-wheuton*	DhRr 6	114-116 N, 3-5W, 40-50 cm	1620-1070 BP	CE 235-1164	*O. keta*	chum	N	P	N	P	M
**BIS29**	*Tum-tumay-wheuton*	DhRr 6	114-116 N, 3-5W, 60-70 cm	~1100 BP	CE 800	—	—	N	N	N	N	—
**BIS3**	*Tum-tumay-wheuton*	DhRr 6	Auger 8 level #15	2269 +/-27 BP	398-210 BCE	*O. gorbuscha*	pink	P	P	P	P	M
**BIS30**	*Tum-tumay-wheuton*	DhRr 6	114-116 N, 3-5W, 60-70 cm	1710 +/-90 BP	CE 94-543	—	—	N	N	N	N	—
**BIS31**	*Tum-tumay-wheuton*	DhRr 6	114-116 N, 3-5W, 60-70 cm	1710 +/-90 BP	CE 94-543	—	—	N	N	N	N	—
**BIS32**	*Tum-tumay-wheuton*	DhRr 6	114-116 N, 3-5W, 60-70 cm	1710 +/-90 BP	CE 94-543	*O. keta*	chum	P	P	P	P	M
**BIS33**	*Tum-tumay-wheuton*	DhRr 6	114-116 N, 3-5W, 60-70 cm	1710 +/-90 BP	CE 94-543	—	—	N	N	N	N	—
**BIS34**	*Tum-tumay-wheuton*	DhRr 6	114-116 N, 3-5W, 60-70 cm	1710 +/-90 BP	CE 94-543	—	—	N	N	N	N	—
**BIS35**	*Tum-tumay-wheuton*	DhRr 6	114-116 N, 3-5W, 60-70 cm	1710 +/-90 BP	CE 94-543	—	chum	N	N	P	N	—
**BIS36**	*Tum-tumay-wheuton*	DhRr 6	114-116 N, 3-5W, 60-70 cm	1710 +/-90 BP	CE 94-543	—	—	N	N	N	N	—
**BIS37**	*Tum-tumay-wheuton*	DhRr 6	114-116 N, 3-5W, 60-70 cm	1710 +/-90 BP	CE 94-543	*O. keta*	chum	N	P	P	P	M
**BIS38**	*Tum-tumay-wheuton*	DhRr 6	114-116 N, 3-5W, 60-70 cm	1710 +/-90 BP	CE 94-543	*O. gorbuscha*	pink	P	N	P	N	F

**BIS39**	*Tum-tumay-wheuton*	DhRr 6	114-116 N, 3-5W, 60-70 cm	1710 +/-90 BP	CE 94-543	*O. gorbuscha*	pink	P	P	P	P	M
**BIS4**	*Tum-tumay-wheuton*	DhRr 6	Auger 8 level #10	1109-1304 BP	CE 659-991	*O. keta*	chum	P	P	P	P	M
**BIS40**	*Tum-tumay-wheuton*	DhRr 6	114-116 N, 3-5W, 60-70 cm	1710 +/-90 BP	CE 94-543	—	—	N	N	N	N	—
**BIS41**	*Tum-tumay-wheuton*	DhRr 6	114-116 N, 3-5W, 60-70 cm	1710 +/-90 BP	CE 94-543	—	—	N	N	N	N	—
**BIS42**	*Tum-tumay-wheuton*	DhRr 6	114-116 N, 3-5W, 60-70 cm	1710 +/-90 BP	CE 94-543	—	—	N	N	N	N	—
**BIS43**	*Tum-tumay-wheuton*	DhRr 6	114-116 N, 3-5W, 60-70 cm	1710 +/-90 BP	CE 94-543	*O. keta*	chum	P	N	P	N	F
**BIS44**	*Tum-tumay-wheuton*	DhRr 6	114-116 N, 3-5W, 60-70 cm	1710 +/-90 BP	CE 94-543	*O. keta*	chum	P	N	N	N	—
**BIS45**	*Tum-tumay-wheuton*	DhRr 6	114-116 N, 3-5W, 60-70 cm	1710 +/-90 BP	CE 94-543	—	—	N	N	N	N	—
**BIS46**	*Tum-tumay-wheuton*	DhRr 6	114-116 N, 3-5W, 60-70 cm	1710 +/-90 BP	CE 94-543	*O. keta*	chum	P	P	P	P	M
**BIS47**	*Tum-tumay-wheuton*	DhRr 6	114-116 N, 3-5W, 60-70 cm	1710 +/-90 BP	CE 94-543	—	—	N	N	N	N	—
**BIS48**	*Tum-tumay-wheuton*	DhRr 6	114-116 N, 3-5W, 60-70 cm	1710 +/-90 BP	CE 94-543	—	—	N	N	N	N	—
**BIS49**	*Tum-tumay-wheuton*	DhRr 6	112-114 N, 3-5W, 120-130 cm	1710 +/-90 BP	CE 94-543	*O. keta*	chum	P	N	P	N	F
**BIS5**	*Tum-tumay-wheuton*	DhRr 6	Auger 8 level #10	1109-1304 BP	CE 659-991	*O. nerka*	sockeye	P	N	P	N	F
**BIS50**	*Tum-tumay-wheuton*	DhRr 6	112-114 N, 3-5W, 120-130 cm	1710 +/-90 BP	CE 94-543	*O. gorbuscha*	pink	P	P	P	P	M
**BIS51**	*Tum-tumay-wheuton*	DhRr 6	112-114 N, 3-5W, 120-130 cm	1710 +/-90 BP	CE 94-543	*O. keta*	chum	P	P	P	N	M
**BIS52**	*Tum-tumay-wheuton*	DhRr 6	112-114 N, 3-5W, 120-130 cm	1710 +/-90 BP	CE 94-543	*O. tshawytscha*	Chinook	P	N	P	N	F
**BIS53**	*Tum-tumay-wheuton*	DhRr 6	112-114 N, 3-5W, 120-130 cm	1710 +/-90 BP	CE 94-543	O. keta	chum	P	P	P	P	M
**BIS54**	*Tum-tumay-wheuton*	DhRr 6	112-114 N, 3-5W, 120-130 cm	1710 +/-90 BP	CE 94-543	O. keta	chum	P	N	P	N	F

**BIS55**	*Tum-tumay-wheuton*	DhRr 6	112-114 N, 3-5W, 120-130 cm	1710 +/-90 BP	CE 94-543	*O. keta*	chum	P	P	P	P	M
**BIS56**	*Tum-tumay-wheuton*	DhRr 6	112-114 N, 3-5W, 120-130 cm	1710 +/-90 BP	CE 94-543	*O. keta*	chum	P	P	P	P	M
**BIS57**	*Tum-tumay-wheuton*	DhRr 6	112-114 N, 3-5W, 120-130 cm	1710 +/-90 BP	CE 94-543	*O. keta*	chum	P	P	P	N	M
**BIS58**	*Tum-tumay-wheuton*	DhRr 6	112-114 N, 3-5W, 120-130 cm	1710 +/-90 BP	CE 94-543	*O. keta*	chum	P	P	P	P	M
**BIS59**	*Tum-tumay-wheuton*	DhRr 6	112-114 N, 3-5W, 120-130 cm	1710 +/-90 BP	CE 94-543	*O. keta*	chum	P	P	P	P	M
**BIS6**	*Tum-tumay-wheuton*	DhRr 6	Auger 8 level #10	1109-1304 BP	CE 659-991	*O. gorbuscha*	pink	P	P	P	P	M
**BIS60**	*Tum-tumay-wheuton*	DhRr 6	112-114 N, 3-5W, 120-130 cm	1710 +/-90 BP	CE 94-543	*O. keta*	chum	P	P	N	P	M
**BIS61**	*Tum-tumay-wheuton*	DhRr 6	112-114 N, 3-5W, 120-130 cm	1710 +/-90 BP	CE 94-543	*O. keta*	chum	P	P	P	P	M
**BIS62**	*Tum-tumay-wheuton*	DhRr 6	112-114 N, 3-5W, 120-130 cm	1710 +/-90 BP	CE 94-543	*O. keta*	chum	P	N	P	N	F
**BIS63**	*Tum-tumay-wheuton*	DhRr 6	112-114 N, 3-5W, 120-130 cm	1710 +/-90 BP	CE 94-543	*O. keta*	chum	P	N	P	N	F
**BIS64**	*Tum-tumay-wheuton*	DhRr 6	112-114 N, 3-5W, 120-130 cm	1710 +/-90 BP	CE 94-543	*O. keta*	chum	P	P	P	N	M
**BIS65**	*Tum-tumay-wheuton*	DhRr 6	112-114 N, 3-5W, 120-130 cm	1710 +/-90 BP	CE 94-543	*O. keta*	chum	P	N	P	N	F
**BIS66**	*Tum-tumay-wheuton*	DhRr 6	112-114 N, 3-5W, 120-130 cm	1710 +/-90 BP	CE 94-543	*O. keta*	chum	P	P	P	N	M
**BIS67**	*Tum-tumay-wheuton*	DhRr 6	112-114 N, 3-5W, 120-130 cm	1710 +/-90 BP	CE 94-543	*O. keta*	chum	P	P	P	P	M
**BIS68**	*Tum-tumay-wheuton*	DhRr 6	112-114 N, 3-5W, 120-130 cm	1710 +/-90 BP	CE 94-543	*O. keta*	chum	P	P	P	N	M
**BIS69**	–	DhRr 22	EU1, strat III, 55-60 cm	1761 BP +/- 24 BP	CE 180-380	*O. keta*	chum	P	P	P	P	M
**BIS7**	*Tum-tumay-wheuton*	DhRr 6	Auger 8 level #7	1174-1109 BP	CE 728-991	*O. gorbuscha*	pink	P	N	P	N	F
**BIS70**	–	DhRr 22	EU1, strat III, 55-60 cm	1761 BP +/- 24 BP	CE 180-380	*O. keta*	chum	P	P	P	P	M

**BIS71**	–	DhRr 22	EU1, strat III, 55-60 cm	1761 BP +/- 24 BP	CE 180-380	*O. keta*	chum	P	P	P	P	M
**BIS72**	–	DhRr 22	EU1, layer 20-25cm	1761 BP +/- 24 BP	CE 180-380	*O. keta*	chum	P	P	P	P	M
**BIS73**	–	DhRr 22	EU1, strat III, 60-65 cm	1761 BP +/- 24 BP	CE 180-380	*O. nerka*	sockeye	P	N	P	N	F
**BIS74**	–	DhRr 22	EU1, strat III, 60-65 cm	1761 BP +/- 24 BP	CE 180-380	*O. keta*	chum	P	P	P	P	M
**BIS75**	–	DhRr 22	EU1, strat III, 40-45 cm	1761 BP +/- 24 BP	CE 180-380	*O. keta*	chum	P	P	P	P	M
**BIS76**	–	DhRr 22	EU1, 65-70cm	1761 BP +/- 24 BP	CE 180-380	*O. keta*	chum	P	P	P	P	M
**BIS77**	*Say-umiton*	DhRr 18	484.75N, 498 E, layer 9 level 1	958 +/- 28 BP	CE 1025-1155	*O. nerka*	sockeye	P	N	P	N	F
**BIS78**	*Say-umiton*	DhRr 18	484.75N, 498 E, layer 9 level 1	958 +/- 28 BP	CE 1025-1155	*O. keta*	chum	P	P	P	P	M
**BIS79**	*Say-umiton*	DhRr 18	484.75N, 498 E, layer 9 level 1	958 +/- 28 BP	CE 1025-1155	*O. keta*	chum	P	N	P	N	F
**BIS8**	*Tum-tumay-wheuton*	DhRr 6	Auger 8 level #7	1174-1109 BP	CE 728-991	*O. gorbuscha*	pink	P	N	P	N	F
**BIS80**	*Say-umiton*	DhRr 18	484.75N, 498 E, layer 9 level 1	958 +/- 28 BP	CE 1025-1155	*O. keta*	chum	P	P	P	P	M
**BIS81**	*Say-umiton*	DhRr 18	484.75N, 498 E, layer 9 level 1	958 +/- 28 BP	CE 1025-1155	*O. keta*	chum	P	P	P	N	M
**BIS82**	*Say-umiton*	DhRr 18	484.75N, 498 E, layer 9 level 1	958 +/- 28 BP	CE 1025-1155	*O. keta*	chum	P	P	P	P	M
**BIS83**	*Say-umiton*	DhRr 18	484.75N, 498 E, layer 9 level 1	958 +/- 28 BP	CE 1025-1155	*O. keta*	chum	P	N	P	N	F
**BIS84**	*Say-umiton*	DhRr 18	484.75N, 498 E, layer 9 level 1	958 +/- 28 BP	CE 1025-1155	*O. keta*	chum	P	P	P	P	M
**BIS85**	*Say-umiton*	DhRr 18	484.75N, 498 E, layer 9 level 1	958 +/- 28 BP	CE 1025-1155	*O. keta*	chum	P	P	P	P	M
**BIS86**	*Say-umiton*	DhRr 18	484.75N, 498 E, layer 9 level 1	958 +/- 28 BP	CE 1025-1155	*O. keta*	chum	P	N	P	N	F
**BIS87**	*Say-umiton*	DhRr 18	484.75N, 496 E, layer 8, level 2	958 +/- 39 BP	CE 999-1168	*O. keta*	chum	P	N	P	N	F
**BIS88**	*Say-umiton*	DhRr 18	484.75N, 496 E, layer 8, level 2	958 +/- 39 BP	CE 999-1168	*O. keta*	chum	P	N	P	N	F

**BIS89**	*Say-umiton*	DhRr 18	484.75N, 496 E, layer 8, level 2	958 +/-39 BP	CE 999-1168	*O. keta*	chum	P	N	P	N	F
**BIS9**	*Tum-tumay-wheuton*	DhRr 6	114-116 N, 3-5W, 40-50 cm	1620-1070 BP	CE 235-1164	*O. keta*	chum	P	N	P	P	M
**BIS90**	*Say-umiton*	DhRr 18	484.75N, 496 E, layer 8, level 2	958 +/-39 BP	CE 999-1168	*O. keta*	chum	P	N	P	N	F
**BIS91**	*Say-umiton*	DhRr 18	484.75N, 496 E, layer 8, level 2	958 +/-39 BP	CE 999-1168	*O. keta*	chum	P	P	P	P	M
**BIS92**	*Say-umiton*	DhRr 18	484.75N, 496 E, layer 8, level 2	958 +/-39 BP	CE 999-1168	*O. keta*	chum	P	P	P	P	M
**BIS93**	*Say-umiton*	DhRr 18	484.75N, 496 E, layer 8, level 2	958 +/-39 BP	CE 999-1168	*O. keta*	chum	P	N	P	N	F
**BIS94**	*Say-umiton*	DhRr 18	484.75N, 496 E, layer 8, level 2	958 +/-39 BP	CE 999-1168	*O. keta*	chum	P	N	P	N	F
**BIS95**	*Say-umiton*	DhRr 18	484.75N, 496 E, layer 8, level 2	958 +/-39 BP	CE 999-1168	*O. keta*	chum	P	P	P	P	M
**BIS96**	*Say-umiton*	DhRr 18	484.75N, 496 E, layer 8, level 2	958 +/-39 BP	CE 999-1168	*O. keta*	chum	P	P	P	P	M
**BIS97**	*Say-ma-mit*	DhRq 1	10-12s, 35-36w, 60-70 cm	1774 +/-67 BP	CE 86-409	*O. keta*	chum	P	P	P	P	M
**BIS98**	*Say-ma-mit*	DhRq 1	10-12s, 35-36w, 60-70 cm	1774 +/-67 BP	CE 86-409	*O. keta*	chum	P	N	P	N	F
**BIS99**	*Say-ma-mit*	DhRq 1	10-12s, 35-36w, 60-70 cm	1774 +/-67 BP	CE 86-409	—	—	N	N	N	N	—
**ST10**	*Say-umiton*	DhRr 18	484.75N, 496 E, layer 8, level 2	958 +/-39 BP	CE 999-1168	*O. keta*	chum	P	P	P	P	M
**ST106**	*Say-umiton*	DhRr 18	484.75N, 498 E, layer 9 level 1	958 +/-28 BP	CE 1025-1155	*O. keta*	chum	P	P	P	P	M
**ST18**	*Say-umiton*	DhRr 18	484.75N, 496 E, layer 8, level 2	958 +/-39 BP	CE 999-1168	*O. keta*	chum	P	N	P	N	F
**ST24**	*Say-umiton*	DhRr 18	484.75N, 496 E, layer 8, level 2	958 +/-39 BP	CE 999-1168	*O. keta*	chum	P	P	P	P	M
**ST244**	*Say-umiton*	DhRr 18	484.75N, 498 E, layer 9 level 1	958 +/-28 BP	CE 1025-1155	*O. gorbuscha*	pink	P	P	P	P	M

**ST28**	*Say-umiton*	DhRr 18	484.75N, 496 E, layer 8, level 2	958 +/- 39 BP	CE 999-1168	*O. keta*	chum	P	P	P	P	M
**ST291**	*Say-umiton*	DhRr 18	484.75N, 498 E, layer 9 level 1	958 +/- 28 BP	CE 1025-1155	*O. keta*	chum	P	N	P	N	F
**ST4**	*Say-umiton*	DhRr 18	484.75N, 496 E, layer 8, level 2	958 +/- 39 BP	CE 999-1168	*O. keta*	chum	P	P	P	P	M
**ST531**	*Say-umiton*	DhRr 18	484.75N, 498 E, layer 9 level 1	958 +/- 28 BP	CE 1025-1155	*O. gorbuscha*	pink	P	P	P	P	M
**ST560**	*Say-umiton*	DhRr 18	484.75N, 498 E, layer 9 level 1	958 +/- 28 BP	CE 1025-1155	*O. keta*	chum	P	N	P	N	F
**ST7**	*Say-umiton*	DhRr 18	484.75N, 496 E, layer 8, level 2	958 +/- 39 BP	CE 999-1168	*O. keta*	chum	P	N	P	N	F

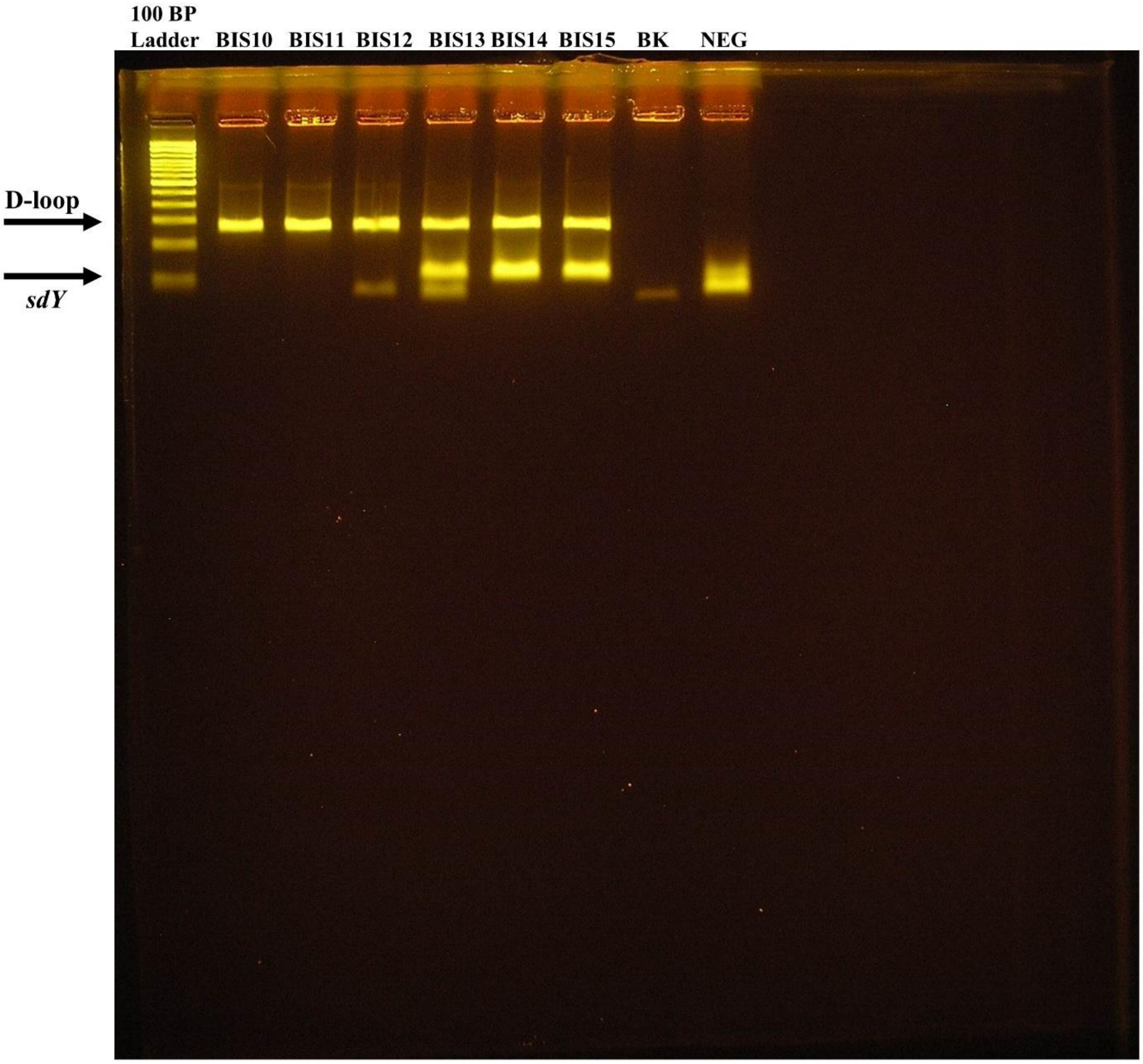

Supplementary Figure 1. Electrophoresis gel showing the results of the D-loop/*sdY* PCR sex identification assay for five of the analyzed Pacific salmon (*Oncorhynchus* spp.) samples (BIS#). The approximate positions of the internal positive control (D-loop) and *sdY* amplicons are indicated by the labelled arrows. BK denotes the blank extraction control processed alongside the samples. NEG denotes the negative PCR control. The 100 bp ladder is from Invitrogen (Vilnius, LT). PCR products were pre-stained with SYBR Green I (Invitrogen, Eugene, OR, USA).

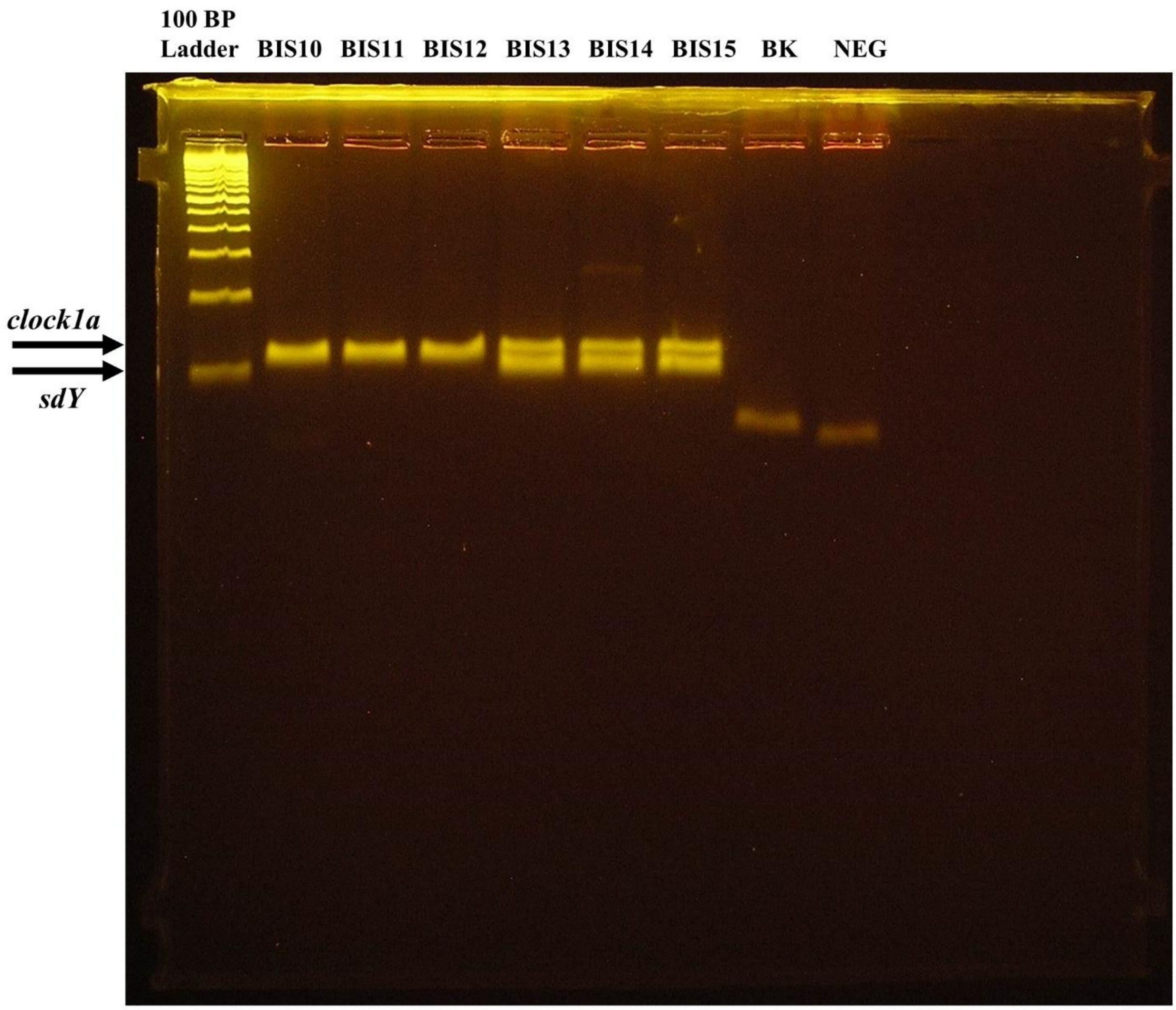

Supplementary Figure 2. Electrophoresis gel showing the results of the *clock1a*/*sdY* PCR sex identification assay for five of the analyzed Pacific salmon (*Oncorhynchus* spp.) samples (BIS#). The approximate positions of the internal positive control (*clock1a*) and *sdY* amplicons are indicated by the labelled arrows. BK denotes the blank extraction control processed alongside the samples. NEG denotes the negative PCR control. The 100 bp ladder is from Invitrogen (Vilnius, LT). PCR products were pre-stained with SYBR Green I (Invitrogen, Eugene, OR, USA).
